# High-Risk Patient Refusals in the Prehospital Setting—Clinical and Legal Considerations

**DOI:** 10.1016/j.acepjo.2025.100083

**Published:** 2025-03-05

**Authors:** Bryan McNeilly, W. Ann Maggiore, Mat Goebel, Michael O’Brien, Nicholas Cozzi, Navin Ariyaprakai, Thomas Lardaro, Michael Weinstock

**Affiliations:** 1 Department of Emergency Medicine, University of Washington, Seattle, Washington, USA; 2 Department of Emergency Medicine, University of New Mexico, Albuquerque, New Mexico, USA; 3 Department of Emergency Medicine, Mercy Medical Center - Trinity Health of New England, Springfield, Massachusetts, USA; 4 Department of Emergency Medicine, University at Buffalo, Buffalo, New York, USA; 5 Department of Emergency Medicine, Rush University Medical Center, Chicago, Illinois, USA; 6 Department of Emergency Medicine, Rutgers Health, Newark, New Jersey, USA; 7 Department of Emergency Medicine, Yale School of Medicine, New Haven, Connecticut, USA; 8 Department of Emergency Medicine, The Wexner Medical Center at the Ohio State University, Columbus, Ohio, USA

**Keywords:** high-risk refusal, medical decision making capacity, EMS transports, EMS left on scene, behavioral health emergencies, against medical advice, transport refusal

## Abstract

When emergency medical service (EMS) arrives on the scene, it is estimated that patients end up declining treatment and/or transport around 5% to 10% of the time. When a patient is suspected of having a life-threatening emergency and declines care, these cases are deemed “high-risk refusals,” as the subsequent delay in treatment drastically increases the risk of morbidity and mortality, as well as the legal risk for all those involved. These cases warrant careful consideration and deliberate training, not only among EMS professionals and EMS medical directors but also among any in-hospital physicians providing online medical oversight. To mitigate the risk associated with high-risk refusals, EMS systems and emergency departments should have standardized policies and guidelines in place for managing these cases, just as with any other complex, high-risk procedure. State statutes should be referenced and ideally legal counsel should be consulted when developing these guidelines. Checklists and quick references are recommended, and calling online medical supervision should be strongly encouraged. EMS medical directors should also routinely review high-risk refusal charts and include these cases in their ongoing quality improvement and quality assessment efforts.

## 1 Introduction

It is estimated that patients end up declining emergency medical service (EMS) treatment and/or transport around 5% to 10% of the time after an emergency call is placed.^1^^,^^2^ When patients have only minor illnesses or injuries, these refusals are relatively low risk. However, when a patient is suspected of having a life-threatening emergency and declines care, these cases are deemed “high-risk refusals,” as the subsequent delay in treatment drastically increases the risk of morbidity and mortality. High-risk refusals are also accompanied by increased legal risk, both at the individual and organizational level. Knowing how to properly manage high-risk refusals is critical not only for EMS personnel and EMS medical directors but also for in-hospital physicians who provide online medical oversight for EMS personnel.

Many systems still do not have a protocol in place for managing high-risk refusals.^3^ Even in systems where a policy is in place, documentation may be inadequate. A recent study of refusals found that 32% did not have documentation regarding the patient’s decision-making capacity or underlying conditions that could have impaired their capacity, such as alcohol consumption, vital sign abnormalities, or diminished Glasgow Coma Scale (GCS) scores.^3^ Unfortunately, despite the complexity and nuance involved in handling high-risk refusals in the prehospital setting, there has been little guidance published on this topic to date. This review provides an overview of important clinical and medicolegal considerations when managing high-risk patient refusals in the prehospital setting.

## 2 General Legal Considerations

The primary challenge in navigating high-risk refusals is striking a balance between respecting a patient’s autonomy and ensuring that they have the information and capacity to make a medical decision. The term capacity is often confused with competency. Competency is a legal evaluation performed by a court of law. In contrast, medical decision-making capacity is determined by medical professionals. In general, capacity in the health care setting is defined in terms of 4 criteria: (1) understanding, (2) appreciation, (3) reasoning, and (4) expression of choice.^4^^,^^5^

• Understanding refers to the individual’s ability to comprehend the information that is being discussed regarding their condition, as well as the potential risks and benefits associated with receiving treatment and foregoing treatment.
• Appreciation refers to the patient’s ability to apply the information being discussed to their situation, ie, how the patient’s choices regarding their medical care could alter their future health and functioning.
• Reasoning refers to whether the patient is arriving at their conclusion rationally.
• Expression of choice refers to the patient’s ability to communicate their decision in a manner that is both clear and consistent.

While assessing these 4 components, it is critical to maintain a broad differential of factors that could impact the patient’s responses, such as language barriers, physical injury, medical illness, intoxication, behavioral health conditions, and cognitive impairment.

High-risk refusals are accompanied by litigious risk from opposing directions. Violating a patient’s autonomy and transporting or providing care against their wishes can be judged just as harshly as incorrectly estimating a patient’s capacity and missing a critical illness. The ruling by Supreme Court Justice Benjamin Cardozo from the case of Schloendorff *v.* New York Hospital, 105 NE 92 (1914), is the basis for many of the decisions made in legal cases dealing with medical consent: *“*Every human being of adult years and a sound mind has the right to decide what shall be done with his own body...This is true except in cases of emergency where the patient is unconscious and where it is necessary to operate before consent can be obtained.”^6^

The opposing litigious risks of violating a patient’s autonomy versus failing to recognize their impaired decision-making capacity are part of why high-risk refusals are so difficult to navigate. As such, these cases warrant the same level of medical oversight and standardized procedural guidance that is given to any of the other complex interventions that EMS performs.

EMS personnel should also be encouraged to utilize online medical oversight whenever possible, as there is some data to suggest that doing so may be associated with an increased likelihood of transport in high-risk patients.^7^ If there is any remaining uncertainty regarding a patient’s capacity even after contacting online medical oversight, EMS should do what they believe to be in the patient’s best interest and document their rationale for doubting the patient’s capacity.^8^ At least one state has legislation authorizing EMS to transport patients against their will in specific situations and limits liability for involuntary transports.^9^ Courts will tend to rule in favor of health care professionals who act in good faith on what is perceived as the best interests of their patient at the time and will tend to rule against health care professionals when there is uncertainty about the patient’s capacity.^10^

## 3 Minors

All states define an age of legal adulthood, termed the age of majority. Typically, this is set at 18, however, this can vary from state to state. Persons younger than the age of majority are considered minors and must be under the care of a parent or guardian. Therefore, a minor has no legal standing, and thus cannot consent to, nor refuse, care from EMS.^11^ An exception to this rule exists for minors who are emancipated. The legal criteria are different in each state, but most states require a minor to be married, financially independent from parents, or active duty military, to be considered emancipated. Some states also consider minors to be emancipated if they are pregnant or are the parents of a child.

In cases involving minors, all efforts should be made to contact a parent or legal guardian to obtain consent without compromising the patient’s care. If parents cannot be contacted in a timely fashion, a person standing in loco parentis may also consent to the care of a minor. In loco parentis is a legal term that means “in place of a parent.” This may be a relative, a teacher, or another person close to the child. If a minor needs immediate care and efforts to locate a parent, guardian, or person in loco parentis are unsuccessful, the patient should be transported to an appropriate facility, and the details surrounding consent can be addressed at a later point in time.

It is considered a crime for a parent or guardian to refuse necessary medical treatment for a minor. In cases where there is reasonable concern for a time-sensitive or life-threatening condition, the patient should be transported regardless of the parent, or patient’s expressed wishes. In these cases, it may be necessary to involve law enforcement to facilitate safely treating and transporting the patient. The use of law enforcement should also be considered in any case where a patient is judged to lack capacity and is aggressively refusing care.

## 3 Mental Health and Psychiatric Emergencies

Patients who are suffering from mental health issues present a unique challenge. Critically, patients with mental health conditions often retain the capacity to make reasoned medical decisions. For example, one study estimated that half of patients hospitalized with schizophrenia could provide informed consent.^12^ However, in the presence of an acute mental health care exacerbation, it can often be difficult for medical professionals to assess a patient’s medical decision-making capacity.

The case *Rivers v. Katz* established grounds for treating patients suffering from acute psychiatric emergencies if the patient poses an immediate or substantial threat to themselves or others.^13^ The challenge then becomes defining and identifying an acute psychiatric emergency and a substantial threat. The courts have used broad and vague definitions for psychiatric emergencies, such as the “occurrence or serious threat of extreme violence, personal injury, or attempted suicide” and the “necessity of preventing immediate, substantial, and irreversible deterioration of a serious mental illness…in which even the smallest of delays would be intolerable.”^14^

Cases where a patient presents with suicidal ideation but may appear to possess decisional capacity can be particularly challenging. In these cases, there is an “emergency exception” to the right to refuse care because the patient is posing a threat to themselves or others. Suicidality in an otherwise healthy person can be interpreted as an irrational mental state and is associated with decreases in several domains of decision-making capacity.^15^, ^16^, ^17^ The responsibility of health care professionals to provide compulsory treatment in such instances is an important exception to the fundamental right to make decisions about one’s health.^18^ Similarly, in many instances, EMS personnel may suspend an advanced directive to treat a patient who is unresponsive due to a suicide attempt, as these documents are typically intended to function in the context of incapacitation due to natural illness or accidental injury.^19^

Mental health conditions add an additional degree of complexity to assessing a patient’s ability to refuse transport and can be a legal minefield for the unwary practitioner.^20^ Ideally, in patients suffering from mental health conditions, a brief psychiatric assessment should be performed in addition to a physical examination. The brief psychiatric assessment should include questions regarding whether the patient has been experiencing any audio or visual hallucinations, paranoia, and inquiries about homicidal or suicidal ideation. Any uncertainty regarding the patient’s safety or their intent to harm themselves warrants a higher level of assessment by a trained professional, whether it be a psychiatric team, a behavioral health professional, or an emergency medicine physician. If there is any doubt regarding how to safely manage these cases, EMS professionals should consult online medical oversight.

## 4 Assessing Capacity in the Intoxicated Patient

It is important to note that patients who have been drinking or using illicit drugs may retain capacity. Intoxication exists on a continuum and is not dictated by a laboratory value or diagnostic test. Rather, intoxication is a clinical determination that must be made by a health care professional. Patients with higher tolerance may still possess decision-making capacity despite high blood alcohol levels or large doses of opioids or other psychoactive substances. In contrast, in chronic alcoholics, low blood alcohol levels may result in severe withdrawal and precipitate delirium, which could impair their capacity more than if their blood alcohol levels were elevated.

Cases in which a patient appears clinically intoxicated warrant an elevated index of suspicion for impaired capacity. It is even more critical in these cases to perform a thorough assessment of capacity and complete the documentation of this assessment in a timely manner. Patients who are not deemed to have capacity or whose capacity is in question should be restrained and involuntarily held.^21^ Critically, it is then incumbent on the health care professional who is restraining the patient to ensure that the form of restraint, whether physical or pharmacologic, does not further injure the patient.

## 5 Transient Capacity Secondary to Rapid Reversal of a Medical Emergency

EMS clinicians may encounter patients in the prehospital environment who undergo a rapid reversal of their altered mental state after they receive effective treatment. Two prominent examples include the response seen after the administration of glucose/dextrose in hypoglycemia and naloxone in an opiate overdose.^22^ In the event that a patient regains consciousness and is deemed to have capacity, even if transient, the patient’s autonomy must be respected, including the right to refuse transport or treatment. If the patient again loses capacity in the presence of the clinician, it is appropriate to assume that consent for assistance is implied and standard care should recommence.

## 6 Elderly Patients, Cognitive Decline, and End-of-Life Considerations

Impaired capacity is rare in elderly adults who are otherwise healthy. However, in the presence of acute disease, the rate of incapacity may be as high as 26%.^23^ Underlying cognitive disorders such as dementia do not necessarily impair a patient’s decision-making capacity. As with mental health disorders and intoxication, the presence of cognitive disorders should prompt a thorough assessment and documentation of a patient’s decision-making capacity. However, it is also important to ensure that known cognitive disorders do not bias the assessment of a patient’s capacity and inadvertently result in an unjustified infringement on a patient’s autonomy.

At times EMS may be called on to provide a lift assist for patients with cognitive decline. There is evidence that over 60% of fall patients in an assisted living facility can be treated in place by an EMS crew without transport.^24^ However, there is also literature demonstrating the risk of simply characterizing a response as a “lift assist.”^25^^,^^26^ If there is any doubt as to whether the patient injured themselves in the event or has an acute underlying illness that prompted their initial call for a lift assist—eg, the patient is unable to provide a detailed account due to their cognitive decline or an altered mental status—then it is not sufficient to simply document the call as a lift assist and leave the scene. Instead, EMS personnel must recommend transport to the emergency department for further evaluation. If the patient refuses transport, then EMS personnel must determine whether they lack capacity. If it is deemed that they lack capacity, every effort must be made to determine if there is a Physician Orders for Life-Sustaining Treatment (POLST) or advanced directive present that supports/clarifies the patient’s wishes. If there is not, an attempt should be made to reach the legal next of kin (NOK) or health care surrogate. If all these attempts are unsuccessful, then the patient must be transported against their wishes to the emergency department for further evaluation. Again, early contact with online medical supervision is strongly encouraged.

Similarly, EMS professionals may encounter patients suffering from terminal conditions who refuse transport. If the patient has capacity, their autonomy must be respected. This includes their right to override their own POLST or advanced directive at any time. If the patient is deemed not to have capacity, then EMS personnel should check for legally valid advanced directives and/or physician/clinician orders that detail the scope of acceptable treatment and goals of care.^27^ It is important to note that a valid Do Not Resuscitate (DNR) or POLST form should supersede instructions given to EMS by a health care surrogate or NOK. If these critical documents are missing or invalid and the patient does not have decision-making capacity, EMS clinicians should then consult the patient’s health care surrogate. In cases where a patient has not designated a surrogate, the authority for making medical decisions is passed on to their NOK in a hierarchical fashion that is determined by state statute. Ideally, the NOK hierarchy should be stated in the high-risk refusal guidelines so that they can easily be referenced on the scene. EMS personnel should be encouraged to seek online medical oversight if there is any doubt as to how to proceed in these cases.

In July of 2023, the Uniform Law Commission approved the updated Uniform Health Care Decisions Act.^28^ The Act addresses advance directives as well as powers of attorney for health care and tries to shield health care agents and professionals from liability when they act reasonably and in good faith. It also adds unmarried life partners to the NOK hierarchy.

## 7 Recommendations

To mitigate the risk associated with high-risk refusals, EMS systems should have a policy in place specifically for managing these cases, just as with other complex, high-risk procedures. State statutes should be referenced and ideally legal counsel should be consulted when developing these policies. In addition, online medical oversight should be encouraged whenever possible. It may also be helpful to develop a checklist that EMS personnel can use as they navigate these complicated scenarios (Table 1). Ideally, the checklist would be filled out digitally and attached to the patient’s electronic medical record. If this is not an option, the checklist should also remind medics about the critical details that need to be documented in the patient’s medical record, including a detailed history of present illness (HPI), a full set of vitals, a physical examination, the assessment of the patient’s capacity, and a discussion of any factors that may impact the patient’s capacity (abnormal vitals, intoxication, cognitive decline, etc.). The chart should also contain the names of all the persons that were involved, including any health care surrogate, NOK, the physician providing online medical oversight, and others. It is also strongly recommended that the routine review of high-risk refusal charts be included in medical directors’ ongoing quality assessment and quality improvement efforts. Finally, EMS personnel and physicians providing online medical supervision should receive focused education and training on how to manage these complex cases (Table 2).

**Table 1:** Components of a high-risk refusal checklist.

Chart Section	Critical component
Demographic characteristics	Name, age, DOB, DPOA or guardian if applicable, primary language, and use of Interpreter
History	Toxic exposure Drugs/alcohol Indicators of endocrine/metabolic crisis Trauma Psychiatric/behavioral health complaints
Medications	Antihyperglycemic medications, anticoagulants or antiplatelets, controlled medications, psychiatric medications
Examination	Two complete sets of vitals Blood glucose level EKG Neuro examination Signs of trauma Signs of shock Signs of drug use or toxic exposures
Assessment of patient’s medical condition	Differential for suspected medical condition (eg, in patient with chest pain- ACS/OMI, PE, pneumothorax, aortic dissection, etc) Risks and suspected outcome if patient declines treatment/transport (eg, delayed diagnosis and treatment, permanent organ injury or death)
Information given to patient	Assessment of medical condition, recommended actions (treat/transport), need for additional diagnostics workup and treatment in the ED, consequences of declining treatment/transport
Assessment of patient’s capacity (with sample questions)	Understanding What medical problem are you having right now? What are options are we proposing to help you? Appreciation What could happen if you accept treatment/transport? What could happen to you if you decline treatment/transport? Reasoning Why don’t you want treatment/transport for your condition? What factors are most important to you in making your decision? Communication Given what we have discussed, what would you like to do?
Capacity decision	Patient is deemed to (have or not have) medical decision-making capacity
Online medical control	Document any physician interaction with the patient and/or guidance given to medics. Document date, time, and physician name. If unable to reach, document attempt(s) made and time(s)
Outcome	Treatment, transport, patient declined, patient taken via POV, Police assistance required for physical restraint and patient sedated for safe transport, etc
Counseling	Patient counseled that they can change their mind at any point and call 911 to proceed with treatment/transport or for further assistance.
Other parties involved (by name)	DPOA, NOK, healthcare staff, daycare workers, etc
Quick references	Medical decision-making hierarchy Special considerations, eg, • Hospice/end-of-life • Behavioral health crisis (suicidal or homicidal ideation) • Cognitive impairment • Intoxication • Minors

ACS, acute coronary syndrome; DOB, date of birth; DPOA, durable power of attorney; ED, emergency department; EKG, electrocardiogram; NOK, next of kin; OMI, occlusion myocardial infarction; PE, pulmonary embolism; POV, private owned vechile.

**Table 2:** Summary table.

General legal considerations	- Capacity: ability to understand, appreciate, reason, and express a choice regarding treatment.
	- Competency is a legal term; capacity is a medical term assessed by health care professionals.
	- Court rulings tend to favor EMS when acting in good faith in the patient’s best interest, even against their wishes.
Minors	- Minors (aged under 18 y, varying by state) cannot generally consent or refuse care.
	- Guardians cannot refuse transport if a medical emergency is suspected.
	- EMS should contact a parent/guardian for consent. If unavailable, a person “in loco parentis” (eg, teacher, relative) may consent.
Mental health and psychiatric emergencies	- Mental illness can impair capacity but not always. Psychiatric emergencies are complex, and a patient’s decisional capacity may still be intact.
	- Suicidal ideation, even in capable patients, can trigger an “emergency exception” to refusal.
Assessing capacity in intoxicated patients	- Intoxication can impair capacity, but intoxication does not automatically negate decision-making ability.
	- Intoxicated patients with impaired capacity should be treated with caution, with thorough documentation and assessment of their ability to make decisions.
Transient capacity	- Some patients (eg, hypoglycemia or opioid overdose) may regain decisional capacity rapidly after treatment.
	- If a patient regains capacity, their autonomy must be respected, but if they lose capacity again, standard care should resume.
Assessing capacity in elderly patients	- Cognitive decline, such as dementia, does not always impair capacity.
	- For patients with cognitive decline who refuse transport, EMS must assess capacity focusing on the patient’s current ability to make decisions, without bias due to their existing diagnosis, and transport for further evaluation if needed.
End-of-life considerations	- Respect for autonomy is paramount in terminally ill patients refusing transport. If the patient lacks capacity, EMS should consult DNR or POLST forms, healthcare surrogates, or family, and contact online medical direction.
	- DNR or POLST forms take precedence over family members’ wishes; documentation and online medical consultation are critical.
Conclusions and recommendations	- EMS systems should have formal protocols for managing high-risk refusals, including a checklist for assessing capacity, contacting medical direction, and documenting key elements.
	- Use online medical direction when in doubt, as it may improve patient outcomes.

EMS, emergency medical service; POLST, Physician Orders for Life-Sustaining Treatment.

## Funding and Support

By *JACEP Open* policy, all authors are required to disclose any and all commercial, financial, and other relationships in any way related to the subject of this article as per ICMJE conflict of interest guidelines (see www.icmje.org). The authors have stated that no such relationships exist.

## Conflict of Interest

The article was written on behalf of the American College of Emergency Physicians Academic Affairs Committee.
